# Neural Processing of Narratives: From Individual Processing to Viral Propagation

**DOI:** 10.3389/fnhum.2020.00253

**Published:** 2020-06-26

**Authors:** Iiro P. Jääskeläinen, Vasily Klucharev, Ksenia Panidi, Anna N. Shestakova

**Affiliations:** ^1^Brain and Mind Laboratory, Department of Neuroscience and Biomedical Engineering, Aalto University School of Science, Espoo, Finland; ^2^International Laboratory of Social Neurobiology, Institute of Cognitive Neuroscience, National Research University Higher School of Economics, Moscow, Russia

**Keywords:** narratives, electroencephalography, functional magnetic resonance imaging, brain, inter-subject correlation, attention, emotions, decision-making

## Abstract

Narratives, in the form of, e.g., written stories, mouth-to-mouth accounts, audiobooks, fiction movies, and media-feeds, powerfully shape the perception of reality and widely influence human decision-making. In this review, we describe findings from recent neuroimaging studies unraveling how narratives influence the human brain, thus shaping perception, cognition, emotions, and decision-making. It appears that narrative sense-making relies on default-mode network (DMN) structures of the brain, especially precuneus. Activity in precuneus further seems to differ for fictitious vs. real narratives. Notably, high inter-subject correlation (ISC) of brain activity during narrative processing seems to predict the efficacy of a narrative. Factors that enhance the ISC of brain activity during narratives include higher levels of attention, emotional arousal, and negative emotional valence. Higher levels of attentional suspense seem to co-vary with activity in the temporoparietal junction, emotional arousal with activity in dorsal attention network, and negative emotional valence with activity in DMN. Lingering after-effects of emotional narratives have been further described in DMN, amygdala, and sensory cortical areas. Finally, inter-individual differences in personality, and cultural-background related analytical and holistic thinking styles, shape ISC of brain activity during narrative perception. Together, these findings offer promising leads for future studies elucidating the effects of narratives on the human brain, and how such effects might predict the efficacy of narratives in modulating decision-making.

## Introduction

Narratives powerfully shape the world-views and decisions of humans. With the term narrative, we mean an (interpretative) account of a situation or series of events (and experiences associated with them) that can be either true or fictitious. Indeed, instead of *homo sapiens*, our species could be called *homo narrans* (Fisher, 1984). The influence of narratives on perception and decision-making has been well described at the behavioral level (Shiller, 2017; Tuckett and Nikolic, 2017; Piotrowski et al., 2019). It has, however, only recently become possible to study how narratives are processed by the brain. As an example of this, comprehension of narrative-level information that accumulates over longer timescales was observed to depend on the default-mode network (DMN) structures of the brain (Nguyen et al., 2019). DMN structures are activated when one engages in vivid daydreaming, thereby constructing scenarios of possible situations, actions, and outcomes for potential future need (Bar, 2009). Understanding of how narratives shape brain activity might offer additional insights, complementing behavioral results, into how narratives shape human perception, cognition, emotions, and decision-making.

**Figure 1:**
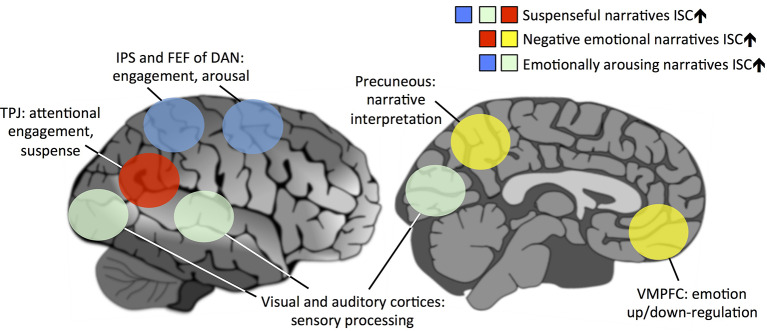
A schematic model showing the brain regions underlying virality of narratives that offers directly testable hypotheses for future studies. We propose that the virality of a narrative centrally depends on how similarly people’s brains are affected by the narrative: the more strongly the narrative synchronizes brain activity across subjects, the more viral it is. However, certain brain areas and networks are more central for this than others. In this model, activation of the temporoparietal junction (TPJ) is centrally associated with higher suspense during a narrative. Attentionally engaging narratives further synchronize the intraparietal sulcus (IPS) and frontal eye field (FEF) of dorsal attention network (DAN) and sensory areas of the brain across subjects. Emotionally arousing narratives also synchronize the DAN and sensory areas. Narratives eliciting negative emotions synchronize the precuneus and ventromedial prefrontal cortex (VMPFC), medial structures of the default-mode network (DMN), thus synchronizing across subjects the narrative interpretation and up/down-regulation of emotional reactions during the narrative, respectively.

The key question in narrative economics is why some narratives are more engaging and effective in “going viral” than others (Shiller, 2017). As examples, the narrative of “*the* *new economy that cannot have downturns*” at the turn of the millennium became a widely held truth, feeding into the .com bubble, and narratives of outbreaks of lethal pandemics, such as H1N1 in 2009, have from time to time swept through societies, widely instilling panic and influencing decisions of individuals and nations alike. Interestingly, some recent neuroimaging studies shed light on the question of why certain narratives prevail and spread (for a summary of studies see Table 1). At least part of the answer seems to lie in how similarly the brains of individuals respond to a narrative (for a schematic model see Figure 1).

**Table 1:** The main research questions, stimuli/paradigms, neuroimaging methods, and main findings, brain areas involved in neuroimaging studies using narratives.

Study	Main research question, stimuli/paradigm	Neuro-imaging method	Main findings, brain areas involved
Altmann et al. (2012)	Written narrative, read as factual vs. fictitious	fMRI	Factual reading involved: RSC, TP, MTG, STG, vSTR, PMC, Cer. Fictitious reading involved: dACC, DLPFC, Precun, IPL, dPCC
Bacha-Trams et al. (2018)	Comparison of ISC of brain activity in subjects with analytical vs. holistic cognitive styles during watching of a drama movie	fMRI	Analytic subjects: AG, STG, TPJ, SPL, Precun, TP, Cer, VMPFC, LING, OC, FP. Holistic subjects: IFG, ACC, FP, Precun, TPJ, Put, VMPFC, AMG
Bacha-Trams et al. (2017)	Comparison of ISC of brain activity when viewing sisters presumed as genetic vs. adopted in a drama movie	fMRI	Genetic: SPL, VMPFC, STG, Put, INS, ITG, MTG, IFG, SFG, IPL, PCG. Adopted: IOG, MOG, Cer, TP, ITG, AG, STG, IFG, PrCG, SFG
Bacha-Trams et al. (2020)	Comparison of ISC of brain activity when viewing a drama movie from moral vs. empathetic perspectives	fMRI	Moral perspective: IOC, MTG, TP, SFG, SPL, Cer, STG. Empathy perspective: MTG, HG, Cer, STG
Bezdek et al. (2017)	Brain regions activated during high-suspense movie clips	fMRI	OTC, IFG, MFG, Cer, LING, MPFC, CC, IPL, STG, SFG, Precun, FG, INS, SMC
Birba et al. (2020)	Written action vs. neutral narrative in subjects’ first and second languages	EEG	Motor cortical activity during a reading of action narratives was reduced when reading in the second language, this effect was predicted by second language proficiency.
Borchardt et al. (2015)	Reduced brain network activity after listening to narratives portraying dismissive childhood experiences	fMRI	SMA, STL, TP, RO, HG, IFG, MPFC, IFL
Borchardt et al. (2018)	Resting-state EEG activity after listening to narratives portraying dismissive childhood experiences	EEG	EEG indices of vigilance decreased after an insecure-attachment narrative
Christoforou et al. (2017)	ISC of EEG during watching of movie trailers	EEG	ISC of EEG during movie trailers predicted box-office performance of the movies
Cohen et al. (2017)	ISC of EEG during watching of engaging vs. non-engaging movie clips	EEG	Higher ISC of EEG during engaging than during non-engaging video clips
Hasson et al. (2009)	Comparison of brain ISC in autistic and neurotypical subjects during movie watching	fMRI	Higher ISC of brain activity in neurotypical than autistic subjects in visual cortical areas
Himichi et al. (2015)	Comparison of positive vs. negative emotional movie clip after-effects on LPFC activity elicited during a ToM task	fNIRS	Lower LPFC activity in the ToM task after negative than a positive movie clip
Jääskeläinen et al. (2016)	Brain regions activated by experienced humorousness during watching of Comedy clips	fMRI	SMG, PrCG, pCC, SLO, ITG, Cer, FC, FP, MTG, SPL, PT, PHG, dACC, LIO, TP, OFC, rACC
Jacob et al. (2018)	Areas mediating between emotional regulation and reactivity networks during anger-eliciting movie clips	fMRI	VMPFC, INS
Ki et al. (2016)	Effects of auditory and audiovisual narrative cohesion on ISC of EEG and attention levels	EEG	Cohesive narratives concomitantly increase ISC of EEG and attention levels
Lahnakoski et al. (2014)	Comparison of detective vs. decorator perspectives during watching of a movie clip	fMRI	PHG, LOC, PPC
Lehne et al. (2015)	Reading narratives and predicting brain activity with higher suspense ratings	fMRI	pSTS, TPJ, IFG, PMC, IFS, MFC
Mano et al. (2009)	Reading of narratives with a spatial perspective on “here now” vs. “there now”	fMRI	TPJ, Pcun
Metz-Lutz et al. (2010)	Areas activated during immersion to theatrical drama	fMRI	BA47, TPJ
Nguyen et al. (2019)	Areas showing ISC of brain activity during the similar interpretation of visual and auditory narratives	fMRI	Precun (posterior medial cortex), DLPFC
Nummenmaa et al. (2012)	ISC of brain activity during emotional movie clips preceded by context descriptions	fMRI	Negative valence: Thal, vSTR, MPFC, ACC, TPJ, Precun, VMPFC. Arousal: visual and somatosensory cortices, IPS, FEF
Nummenmaa et al. (2014a)	ISPS of brain activity during experienced valence and arousal while listening to narratives	fMRI	Negative valence: ACC, Precun, LING. Positive valence: SOG, MFG, MPFC. Negative arousal: MFG, MTG, Precun, LING, MFG. Positive arousal. AG, Cer, SFG, MFG, MOG, AG, STG, MTG
Nummenmaa et al. (2014b)	ISPS of brain activity during simulation of boxers vs. passive viewing of boxing clips	fMRI	Sensory cortices, PrCG, SPL, IPL, STG, MTG, IPS, FEF, LOC
Pichon et al. (2015)	Aftereffects of viewing emotionally negative and positive valenced movie clips on amygdala reactivity to fearful faces	fMRI	AMG
Salmi et al. (2013)	Higher ISC of brain activity during drama-movie watching in neurotypical than high-functioning autistic subjects	fMRI	SMG, LOC, CN, ACC, Precun, PCC, SMA, NAccumb, INS, SMG
Tikka et al. (2018)	IC-based identification of brain areas activated by similarly by a movie and its textual script	fMRI	PreCun, Calc, OMG, OSG, PHG, cuneus, SP, FG, IPC, AG, mCC, FCx, PCG, SMG, MTC, STC, Cer, CN, Thal, SMA, ACC, PCG, IOC
van Marle et al. (2009)	Areas showing increased sensitivity to emotional facial stimuli after acute film-clip induced stress	fMRI	VIS, FFA, AMG
van Marle et al. (2010)	Areas showing increased connectivity with amygdala during resting state following a stressful movie	fMRI	HC, Thal, INS, ACC, STG, Precun, Cer, FG, IFG, PHG, MFG, SFG, mORG, brainstem
Yeshurun et al. (2017)	Listening to an audiobook with *a priori* information about real cheating vs. paranoid jealousy in the story	fMRI	Precun, pCC, TPJ, STS, TP, PMC, DMPFC, VMFPC, HC, Thal

*Abbreviations: ACC, anterior cingulate gyrus; AG, angular gyrus; AMG, amygdala; Calc, calcarine cortex; CC, cingulate cortex; Cer, cerebellum; CN, caudate nucleus; dACC, dorsal anterior cingulate cortex; DLPFC, dorsolateral prefrontal cortex; DMPFC, dorsomedial prefrontal cortex; dPCC, dorsal posterior cingulate cortex; FC, fusiform cortex; FCx, frontal cortex; FEF, frontal eye field; FFA, fusiform face area; FG, fusiform gyrus; FP, frontal pole; HC, hippocampus; HG, hippocampal gyrus; IFG, inferior frontal gyrus; IFL, inferior frontal lobe; IFS, inferior frontal sulcus; INS, insula; IOC, inferior occipital cortex; IOG, inferior occipital gyrus; IPC, inferior parietal cortex; IPL, inferior parietal lobule; IPS, intraparietal sulcus; ITG, inferior temporal gyrus; LPFC, lateral prefrontal cortex; LING, lingual gyrus; LIO, lateral inferior occipital; LOC, lateral occipital cortex; MFC, medial frontal cortex; MOG, middle occipital gyrus; mORG, middle orbital gyrus; MPFC, medial prefrontal cortex; MTC, middle temporal cortex; MTG, middle temporal gyrus; NAccumb, nucleus accumbens; OC, occipital cortex; OFC, orbitofrontal cortex; OTC, occipitotemporal cortex; pCC, posterior cingulate cortex; PCG, post-central gyrus; PrCG, precentral gyrus; PHG, parahippocampal gyrus; PMC, premotor cortex; PPC, posterior parietal cortex; PrCG, precentral gyrus; Precun, precuneous; pSTS, posterior superior temporal sulcus; PT, planum temporale; Put, putamen; rACC, rostral anterior cingulate cortex; RO, Rolandic operculum; RSC, retrosplenial cortex; SFG, superior frontal gyrus; SLO, superior lateral occipital cortex; SMC, sensorimotor cortex; SP, superior parietal cortex; SPL, superior parietal lobule; STC, superior temporal cortex; STG, superior temporal gyrus; STL, superior temporal lobe; Thal, thalamus; TP, temporal pole; TPJ, temporoparietal junction; VIS, visual cortex; VMPFC, ventromedial prefrontal cortex; vSTR, ventral striatum*.

Such similarity is estimated by calculating inter-subject correlations (ISC) of brain activity measured with electroencephalography (EEG) or functional magnetic resonance imaging (fMRI) during narratives. The ISC is a model-free analysis approach wherein, in case of fMRI, the brains of individual subjects are first co-registered, followed by calculation of correlation coefficient of the time series of brain activity in each individual voxel or region-of-interest either across all pairs of subjects or between (sub)groups of subjects (Hasson et al., 2004, 2010; Kauppi et al., 2010). For EEG, the ISC can be calculated between correlated components of the activity of experimental subjects (Dmochowski et al., 2012).

## Higher Inter-Subject Synchrony of Brain Activity During A Narrative Predict Its Virulence

Neuroscience of language has nearly ignored the role of narratives in behavioral adjustments and social influence. On the other hand, neuroscience of social influence primarily focuses on the neural activity of the “communicators” and “receivers” (Falk and Scholz, 2018) and overlooks the role of narratives (persuasive messages) in social communication. Various simplified experimental paradigms, modeling social influence in neuroimaging studies (Klucharev and Shestakova, 2015; Toelch and Dolan, 2015), are poorly suited to investigate natural dynamic persuasive communication. ISC may provide an effective tool to uncover which aspects of natural persuasive messages make them effective.

In a seminal study, the ISC of EEG activity during watching movie trailers predicted the popularity of commercial movies >20 times better than behavioral methods used by the movie industry (Christoforou et al., 2017). This suggests that ISC of EEG activity predicted which movie trailers (i.e., short narratives of the movies) became viral enough to influence people in massive amounts to go and watch the movie. Naturally, the effect of a movie trailer is not the only influencing factor in the commercial success of the corresponding movie. Nonetheless, the strength of the study was in this real-world outcome measure and the robustness of the predictive power. While these results point out the importance of across-subjects synchronization of brain activity for a narrative to become “virulent,” they do not reveal the synchrony of which neurocognitive processes enhance ISC.

Other EEG studies have disclosed that enhanced ISC of EEG activity during meaningful auditory and audiovisual narratives goes hand-in-hand with the attentional state of the subjects (Ki et al., 2016) and that ISC of EEG activity predicts subjects’ voluntary sustained attention to a movie (Cohen et al., 2017). Further, the ISC of EEG activity predicts attentional engagement better than EEG alpha-band power during the narratives (Ki et al., 2016). Thus, a mere increase in vigilance does not explain the higher ISC, but it is rather explained by attentional modulation of narrative processing. Thus, it seems that attentionally engaging narratives boost the ISC of EEG.

EEG does not allow accurate estimation of brain regions that are synchronized across subjects during a contagious narrative. FMRI offers better spatial accuracy. Brain areas consistently reported to support higher levels of suspense during narratives are lateral temporal and inferior parietal areas in and around temporal-parietal junction (TPJ; Metz-Lutz et al., 2010; Lehne et al., 2015; Bezdek et al., 2017). Previous studies have shown that TPJ is a central node in brain networks supporting attentional processing (Corbetta and Shulman, 2002). Thus, boosting of attentional levels *via* recruitment of TPJ might underlie higher levels of suspense during a captivating narrative. However, there are also other mechanisms *via* which narratives synchronize brain activity, including emotions.

## Emotional Valence and Arousal During Narratives Differentially Synchronize The Brain

The emotional impact has been speculated to contribute to narrative contagiousness (Shiller, 2017). Narratives are among the most potent of emotion-eliciting stimuli (Westermann et al., 1996). This becomes even more evident if one understands narratives more broadly, as narratives and movie clips represent the two most potent categories of emotion-eliciting stimuli (Westermann et al., 1996). Even induction of anger often recognized to be challenging, is possible with narrated movie clips as stimuli (Jacob et al., 2018).

Emotions are an important factor in decision-making. The classical economic analysis suggested that individuals make decisions rationally based on utility maximization (von Neumann and Morgenstern, 1947). More recent studies have suggested that emotions influence decision-making in complex environments (Zajonc, 1980; Tuckett and Nikolic, 2017). As another example, emotion-eliciting narratives communicate information about policies to the majority of citizens more effectively than factual-only communications (Piotrowski et al., 2019). Known as the availability heuristic, decision-making under risk is explained better by the ease to recall or imagine a similar event than by the objective probability of a particular event (Folkes, 1988; Kousky, 2010; Bin and Landry, 2013). As a consequence, dramatic and vividly described events disproportionately influence participants’ perception of risk and decisions (Loewenstein et al., 2001). In the somatic-marker model, emotions also play a key role in guiding decision-making (Bechara and Damasio, 2005). Relying on emotions in decision-making might also explain the divergence between public and expert opinions on the risks associated with various events (Lachlan and Spence, 2010). Additionally, emotions have been shown to affect investment decisions (Eberhardt et al., 2019), impulsive behavior in the monetary domain (Lerner et al., 2013), and perception of fairness (Chen and Kamei, 2018). Thus, increased knowledge of how emotional narratives shape brain activity might help explain how emotions modulate decision-making.

Multiple studies have shown increased ISC of brain activity during emotionally stimulating narratives. In one study, emotional movie clips, preceded by contextual descriptions, were presented to experimental subjects during fMRI (Nummenmaa et al., 2012). After fMRI, the subjects dynamically rated their experienced emotional valence and arousal that were used to predict dynamic ISC values. Higher levels of emotional arousal synchronized areas within the dorsal attention network (DAN) and visual-cortical areas (Nummenmaa et al., 2012). This finding is in line with the observations of higher ISC of EEG activity during captivating narratives being due to higher levels of attention (Ki et al., 2016).

Negative emotional valence, in turn, enhanced ISC in DMN structures (Nummenmaa et al., 2012, 2014b). This finding was speculated to reflect the propensity of negative emotions to put human beings in restricted (“fight-or-flight”) processing modes (Nummenmaa et al., 2012). DMN midline structures have been also suggested to support an associative process whereby what one sees or hears rapidly elicits associations that build one’s mental content on a moment-by-moment basis to help make sense and predict the complex world (Bar, 2007). These findings suggest that narratives that contain negative emotions can effectively synchronize the thoughts of individuals. This suggests that negative-emotional narratives have a biological basis for becoming easily “viral.”

Emotional narratives also exert lingering effects on brain activity/responsiveness. Emotional movie clips alter amygdala reactivity (van Marle et al., 2009; Pichon et al., 2015), and sensitize sensory areas of the brain (van Marle et al., 2009). Functional connectivity changes were also reported after emotional narratives and movies (van Marle et al., 2010; Borchardt et al., 2015). EEG findings have suggested that subjects remain aroused after emotional narratives (Borchardt et al., 2018). Finally, negative emotions induced by movie clips inhibited lateral prefrontal cortical activation during a subsequent theory of mind task (Himichi et al., 2015). Together, these findings indicate that emotionally engaging narratives shape subsequent information processing in the brain. This is, however, not limited to emotions elicited by narratives. Non-emotional information also frames or biases the processing of subsequent information, causing subjects to adopt differential perspectives when interpreting a narrative, which is discussed next.

## Subjective Perspective Impacts The Way Narratives Modulate Brain Activity

Contagious narratives are perceived, and exert their impact, in specific contexts (Shiller, 2017). For example, the phrase “*the only thing we have to fear is fear itself*” by President Franklin Roosevelt in 1933 became famous and effective in the particular context of tumultuous world events of the time, amidst which Roosevelt managed to instill the much-needed courage by giving people a specific perspective. In a different context void of threats, the meaning of the phrase would have been quite different. There are neuroimaging studies that have looked at how contextual cues, and perspective that one perceives a given narrative from, shape the processing of narratives in the human brain.

In one study, subjects were informed about genetic vs. adopted relationship between two protagonists before watching a drama movie depicting a moral dilemma (Bacha-Trams et al., 2017). While over 90% of the subjects self-reported that the genetic vs. adopted relationship was not relevant, ISC of brain hemodynamic activity widely differed between the two conditions. When watching the protagonists as genetic sisters (as opposed to adopted) stronger ISC was observed especially in the medial DMN structures. In the reverse contrast, higher ISC was observed in mainly sensory and lateral prefrontal areas of the brain (Bacha-Trams et al., 2017). Thus, even a small differential piece of *a priori* information can shape how the brain processes a subsequent narrative. In another study, subjects were primed to hear an audiobook from two different social perspectives: the protagonist in the story exhibiting unfounded vs. justified jealousy. The subjects showed ISC differences in the DAN, hippocampus, DMN, mirror neuron system, and language processing networks between these two perspective conditions (Yeshurun et al., 2017).

There are also studies where explicit perspective-taking instructions were given to subjects before watching a narrated movie clip during fMRI. Perspectives of a detective vs. an interior decorator modulated activity in DAN and posterior hippocampi, suggesting that these areas control perspective-specific attentional information gathering in naturalistic settings (Lahnakoski et al., 2014). Eye-movements explained these differences only in early visual cortical areas. In another study, perspective-taking with differential knowledge of spatial locations of protagonist and events related to the protagonist activated TPJ and posterior cingulate cortex (pCC) in addition to the mentalizing network of the brain (Mano et al., 2009). Social perspectives requiring empathy vs. moral cognition resulted in patterns of increased ISC in areas supporting empathy and moral cognition, respectively (Bacha-Trams et al., 2020). A finding that is closely related to perspective-taking, reading a passage labeled as fiction activated precuneus more strongly than reading the same passage labeled as factual (Altmann et al., 2012). This might help explain how attributing a given news source as fake vs. real so drastically impacts the interpretation of the news.

Behavioral-economic studies suggest that perspective-taking influences decisions that participants make. In a task where subjects chose between a smaller reward sooner and a larger one later, they expressed less impulsive behavior when they decided for themselves vs. another person (Ziegler and Tunney, 2012). People also take more risks when making decisions for others compared to themselves, mainly due to differences in perception of losses (Sokol-Hessner et al., 2009; Polman and Wu, 2020). Self-other perspective discrepancies in loss aversion correlated with a weaker galvanic skin-conductance response to losses (Sokol-Hessner et al., 2009) as well as greater involvement of brain areas linked to decision making in the loss domain such as anterior insula (Zhang et al., 2019). Differences in self-other behavior have been demonstrated in recent experimental studies concerning various biases, such as anchoring bias or the endowment effect (Ifcher and Zarghamee, 2020). Some studies in the healthcare domain showed the opposite pattern: physicians prefer treatments with higher mortality rates when deciding for themselves, but fewer of them choose to recommend these options to their patients (Ubel et al., 2011; Garcia-Retamero and Galesic, 2012). Neuroimaging findings in turn suggest that subjects are less effectively engaged when deciding on behalf of others, and are thus less affected by psychological biases (Albrecht et al., 2011; Ogawa et al., 2018). Taken together, these findings suggest that during perspective taking the brain flexibly recruits regions and functions based on the cognitive, perceptual, and emotional requirements of the perspective.

## Personality and Cultural Background Factors Shape Narrative Processing in The Brain

Individual differences in personality and cultural background shape processing of narratives in the brain. As examples, persons with autistic features processed narratives more idiosyncratically than neurotypical controls (Hasson et al., 2009; Salmi et al., 2013) and differential patterns of ISC were observed in subjects with analytical vs. holistic cognitive styles (Bacha-Trams et al., 2018). The analytical and holistic cognitive styles are predominant cognitive-perceptual tendencies in Western and Eastern cultures, respectively. For example, holistic individuals concentrate less on objects and view also background, whereas analytical subjects focus on objects. There are, however, also other differences, for example, on the perception of whether different phenomena are interrelated or not (Choi et al., 2007). Together, these results suggest that the efficacy of narratives greatly depends on the personality and cultural background of the recipient. From this, it follows, for example, that a given narrative can become far more virulent in a culturally homogeneous than in a culturally heterogeneous nation.

Diversity in cognitive styles and cultural differences significantly shape also decision-making (Weber and Hsee, 2000). Chinese participants, as representatives of a more collectivist culture, showed less risk-aversion in financial risk-taking than American participants (Weber et al., 1998; Hsee and Weber, 1999). In accordance with cushion hypothesis social networks, rather than nationality, predict risk preference in empirical studies (Schneider et al., 2017; Illiashenko, 2019). Moreover, not only risk attitudes differed between Chinese and more analytical Americans, but Chinese proverbs were more risk-promoting than American proverbs (Weber et al., 1998). This suggests that culture-specific differences in risk-taking are manifested in language. Additionally, Chinese subjects demonstrated less probabilistic thinking compared to English participants which partly explained their higher propensity to gamble (Lau and Ranyard, 2005). Further, Chinese subjects employ a specific method of decision-making called folk-matching decisions: when confronted with a problem, a precedent stored in the form of a story is utilized in decision-making (Yates and Lee, 1996; Weber et al., 2005). It is an interesting open question whether such precedent narratives cause higher ISC of brain activity than other stories and whether this might explain the virulent and influential properties of the narratives.

## Challenges in Neuroimaging Studies of Narrative Processing

Narratives are complex stimuli and it is difficult to discern effects due to the narrative from other aspects of stimulation or language processing. This applies to many of the studies described in this review. Recent studies have controlled for this by showing both the film and its script and inspected commonalities across the two forms of narrative presentation (Tikka et al., 2018) and by obtaining behavioral self-reports on the interpretation of visual and auditory narratives to inform neuroimaging data analysis (Nguyen et al., 2019). These serve as excellent examples of controls that help overcome potential problems. Overall, collecting behavioral self-reports on how narratives are experienced to guide the neuroimaging analysis *via* for example representational similarity analyses helps pinpoint specific narrative-related effects when using complex naturalistic stimuli such as auditory narratives or film clips (Nummenmaa et al., 2014a; Jääskeläinen et al., 2016). This is especially important given there are factors, such as the language proficiency of the subjects, that can modulate the effects of the narrative especially in multi-lingual environments (Birba et al., 2020).

## Conclusions

Narrative economics is an exciting area wherein behavioral findings advance understanding of how narratives influence the financial decision-making of individuals and groups (Shiller, 2017). Narratives robustly shape who we are, how we think and perceive the world, the decisions we make, and how we collectively take risks during financial bubbles and economic crises (Shiller, 2017; Tuckett and Nikolic, 2017; Piotrowski et al., 2019). Recent findings provide insights into how narratives might shape decision-making in the human brain. Enhanced ISC of brain activity seems to co-occur with increased “virality” of narratives. Further, enhanced levels of attention correlate with increased ISC. TPJ activity co-occurs with increased suspense during a narrative. There is increased ISC of brain activity in DAN and sensory areas during emotional arousal, and in DMN structures during negative emotional valence. Besides emotions, contextual cues and explicitly adopted perspectives, as well as personality and cultural background factors shape the processing of narratives in the brain. Finally, recent methodological advances make it possible to isolate the effects of narrative processing in the brain during naturalistic stimulation. Examples of potential application areas include behavioral insights, for example informing governments on the steering of human behavior, studies into health effects of narratives at the population level, clinical research on narrative processing deficits in certain psychiatric and neurological disorders, as well as facilitation of the development of efficient audiovisual educational materials.

## Author Contributions

IJ, VK, KP, and AS contributed to the literature search, writing of the manuscript and approved the final manuscript.

## Conflict of Interest

The authors declare that the research was conducted in the absence of any commercial or financial relationships that could be construed as a potential conflict of interest.
